# Supplementary material for: Time Makes Space: Emergence of Place Fields in Networks Encoding Temporally Continuous Sensory Experiences
Source: bioRxiv. 2025 Jan 29:2024.08.11.607484. Originally published 2024 Aug 12. Preprint. [Version 2] doi: 10.1101/2024.08.11.607484 (PMC11343115; doi:10.1101/2024.08.11.607484)
Supplement: Supplementary file 1 [file NIHPP2024.08.11.607484v2-supplement-1.pdf]

bioRxiv preprint doi: https://doi.org/10.1101/2024.08.11.607484; this version posted January 29, 2025. The copyright holder for this preprint (which was not certified by peer review) is the author/funder, who has granted bioRxiv a license to display the preprint in perpetuity. It is made available under aCC-BY 4.0 International license.

Supplemental Materials

1 Training details

1.1 Simulated trials and the artificial agent

An artificial agent explores simulated room(s) defined by fixed WSM signals, as defined in Section 2.2. The agent updates its behavior and samples sensory experience every 50ms. To realistically model rodent movement trajectories, we simulate a random walk, where artificial agents explore the room in a trajectory with random small drifts representing an animal's exploration interest: at every timestep, the agent randomly changes its movement speed and direction with a small probability, 0.2 and 0.3, respectively. The change in speed is randomly chosen from an $\mathcal{N}(5, 1)$ cm/s normal distribution, and the drift direction is randomly chosen from $\mathcal{N}(0, 0.05)$ rad/s.

1.2 Training Recurrent Autoencoder (RAE)

The simulated artificial agent gathers experience vectors along its trajectory. We train the RAE on 1s experience segments with a batch size of 500, sampled from a 300s 'decaying' distribution of recent events to encourage the network update in consideration of an extended history of events. For an event that is t seconds away from the current time step, the probability of it being sampled is:

$$P(t) = \frac{\left(\frac{T_s - t}{T_s}\right)^{\alpha} + \beta}{\sum_{j=1}^{T_s} \left(\frac{j}{T_s}\right)^{\alpha} + \beta} \tag{1}$$

Where T_s is the width of the sampling window, $\alpha = 3$ and $\beta = 0.5$.

Our RAE contains three layers: The first and the third layers represent the signals being projected to and read out from CA3, respectively. The hidden layer, being fully connected by recurrent connections, corresponds to CA3. The hidden-layer states, which model activation potentials of neurons, are governed by the following continuous-time equation:

$$\tau \frac{d\mathbf{v}_t}{dt} = -\mathbf{v}_t + \mathbf{W}^{rc} f(\mathbf{v}_t) + \mathbf{W}^{in}\mathbf{e}'_t + \mathbf{b} + \boldsymbol{\eta}_t \tag{2}$$

Here $f(x)$ is an activation function and τ governs the decay. $\mathbf{W}^{in}$ is the connection matrix to the hidden layer, simulating pathways entering the hippocampus. $\mathbf{W}^{rc}$ is the recurrent connectivity matrix emulating CA3 recurrent collaterals. The vector $\mathbf{b}$ is a firing bias term and $\boldsymbol{\eta}_t$ denotes Gaussian pre-activation noise. The variable $\mathbf{v}_t$ models neural activation potentials. Setting $\gamma = \frac{dt}{\tau}$ and adapting to discrete- time gives:

$$\mathbf{v}_{t+1} = \mathbf{v}_t + \Delta\mathbf{v}_t = (1-\gamma)\mathbf{v}_t + \gamma\left[\mathbf{W}^{rc}\mathbf{h}_t + \mathbf{W}^{in}\mathbf{e}'_{t+1} + \mathbf{b} + \boldsymbol{\eta}_{t+1}\right] \tag{3}$$

$$\mathbf{h}_t = f(\mathbf{v}_t) + \boldsymbol{\xi}_t \tag{4}$$

Here we have replaced $f(\mathbf{v}_t)$ in equation (2) with equation (4), where $\mathbf{h}_t$ is the state vector of the hidden layer neurons in Hertz (Hz) and $\boldsymbol{\xi}_t$ is the post-activation noise.

The network is trained with mean-squared error without strict sparseness constraints. The loss function is:

$$\mathcal{L} = \frac{\lambda_{mse}}{D \cdot T \cdot B} \sum_{d,t,b}^{D,T,B} (\hat{e}_{d,t,b} - e_{d,t,b})^2 + \frac{\lambda_{fr}}{N} \sum_{n}^{N} \left(\sum_{t,b}^{T,B} r_{n,t,b} \right)^2 \tag{5}$$

D is the dimension of the Experience Vector (EV), T measures timesteps within a memory segment, B indicates the number of batches, and N counts nodes in the hidden layer. The term $\hat{e}_{d,t,b}$ is the d-th entry of the reconstructed EV at timestep t in batch b, while $e_{d,t,b}$ is the equivalent entry of the ground truth. The second component of the loss, $r_{n,t,b}$, denotes the firing rate of the n-th neuron at time t within batch b. Unless otherwise stated, we set $\lambda_{mse} = 0$ and $\lambda_{fr} = 200$ for our experiments.

bioRxiv preprint doi: https://doi.org/10.1101/2024.08.11.607484; this version posted January 29, 2025. The copyright holder for this preprint (which was not certified by peer review) is the author/funder, who has granted bioRxiv a license to display the preprint in perpetuity. It is made available under aCC-BY 4.0 International license.

Table 1: Network parameters

Parameter	Description	Value
n_hidden	# hidden layer nodes	1000
InputLayer	Distribution of the input layer	$\mathcal{U}(-\sqrt{k}, \sqrt{k})$, where $k = \frac{1}{\text{in\_features}}$
HiddenLayer	Distribution of the hidden layer	$\mathcal{U}(-\sqrt{k}, \sqrt{k})$, where $k = \frac{1}{\text{n\_hidden}}$
OutputLayer	Distribution of the output layer	$\mathcal{U}(-\sqrt{k}, \sqrt{k})$, where $k = \frac{1}{\text{n\_hidden}}$
dt	Time resolution	50 ms
τ	Time constant	500
$\gamma = \frac{dt}{\tau}$	Decaying factor	0.1
Optimizer	Network optimizer	Adam
learning_rate	The learning rate of RAE	0.0005
λ_{mse}	Coefficient for the mean-squared pattern completion error	1
λ_{fr}	Coefficient for the mean-squared hidden layer firing rates	200

Table 2: Training parameters

Parameter	Description	Value
N_{batch}	Number of episodic memory in each 'bout'	500
warmup_duration	The duration of experience vector before the first trial room (see Fig. 1b)	300 s
masking_ratio	The percentage we use to randomly occlude an arbitrary experience vector	$\mathcal{U}(r_{min}, r_{max})$ $r_{min} = 0$ & $r_{max} = 0.2$
α	The exponential coefficient in equation (1)	3
β	The constant term in equation (1)	0.05
spatial_resolution	How each meter in the real-world is converted into pixels in our simulated rooms	1 cm/pixel
T_w	Width of the sampling window (see Fig. 1 caption)	300 s
T_s	Length of each episodic memory	1 s
Δt	The step size of how much to move the sampling window forward	1 s

1.3 Testing RAE and visualizing hidden layer firing profiles

After training in each trial, we paused parameter updates and "recorded" the network response at every location in the room. The network continued to receive batches of partially occluded experiences with added noise, generated by a random traveling agent, as detailed in the main text. To ensure sufficient data at every location and to average out effects of random occlusions and noise, we conducted 20-minute trials repeated 20 times. Importantly, the obtained spatial maps are robust to both the duration and the number of tests since we paused parameter updates during testing.

During these tests, we recorded the firing rate (post-activation value) of each hidden layer unit at every location. We then averaged the unit's responses at each location across all previous visits. We repeated this process for all locations and hidden layer units to obtain spatial maps after.

2 Measurements and metrics

2.1 Spatial information content

We used the Spatial Information Content (SIC) [51] measured in bits to measure the strength of a cell's place-like property in terms of its spatial selectivity We discretize firing rate maps into M 30cm $\times$ 30cm bins. Each bin has a firing rate and associated probability p_m of the cell firing. The spatial

bioRxiv preprint doi: https://doi.org/10.1101/2024.08.11.607484; this version posted January 29, 2025. The copyright holder for this preprint (which was not certified by peer review) is the author/funder, who has granted bioRxiv a license to display the preprint in perpetuity. It is made available under aCC-BY 4.0 International license.

information content (SIC) is computed as:

$$SIC = \sum_{m=1}^{M} p_m \cdot (\frac{r_m}{\bar{r}}) \cdot log_2(\frac{r_m}{\bar{r}})$$

where $\bar{r}$ is the average firing rate across bins. Bins with $r_m = 0$ are skipped in the sum (to avoid divergences in the log), but are included in the computation of the average rate $\bar{r}$.

2.2 Synapse reorganization score

We quantify synaptic adaptation as the ratio of the Frobenius norm of the change in the weight matrix to the Frobenius norm of the weight matrix prior to update:

$$\frac{||W^{old} - W^{new}||_F}{||W^{old}||_F}$$

Here, W^{old} and W^{new} represent synaptic weight matrices before and after adaptation, respectively.

3 Orthogonal spatial representations

3.1 Measurement of orthogonality

To measure how generated place-like patterns form representations of different rooms, and to what extent these representations are orthogonal, we adopted a methodology similar to that proposed by Alme et al. [21]. At the end of each trial, we converted the 100 × 100 cm firing rate map from a single unit into a 20 × 20 grid by averaging the rates within each bin to obtain a population coding vector for a room. To compute the similarity between two population vectors, X and Y, Alme et al. [21] used the mean dot product:

$$\frac{1}{N \cdot W \cdot H} \sum_{i,j,k}^{N,W,H} X_{i,j,k} Y_{i,j,k}$$

Here N is the number of units, and W and H are the bin dimensions in width and height, respectively. The dot product involves the population vector of a room as a whole. However, to provide a normalized measure of similarity for Fig. 5 in our main paper, we adopted the Pearson correlation. Pearson correlation also uses the population vector of a room as a whole, but normalizes the data to reduce the dominance of highly active units. Thus, the similarity between X and Y is calculated as:

$$\frac{\sum_{i=1}^{N \cdot W \cdot H} (X_i - \overline{X})(Y_i - \overline{Y})}{\sqrt{\sum_{i=1}^{N \cdot W \cdot H} (X_i - \overline{X})^2} \sqrt{\sum_{i=1}^{N \cdot W \cdot H} (Y_i - \overline{Y})^2}}$$

3.2 Drifting place fields and orthogonal representations

To test whether the generated representations can be used to construct cognitive maps for different enclosures, and whether these maps can be maintained over an extended period, we placed the agent in 20 rooms for 30 cycles, resulting in 600 recordings. In the main text, we compared how spatial representations in the last cycle compared to representations from previous cycles. Here, we offer a more comprehensive comparison. Suppl. Fig. 1 presents the cross-comparison of all 600 recordings, showing that recordings from the same room remain correlated even many cycles apart. Only recordings from cycle 1 show a slightly decreased correlation score, due to the incomplete formation of place fields.

As the simulated agent travels through multiple rooms over an increasing number of cycles, we observe that place cells, while reverting to their previous locations upon reentering a familiar room, may exhibit slight shifts in their locations. As the number of cycles between two trials of a single room increases, this discrepancy becomes increasingly pronounced, manifesting as a place field drift. Figure 2 depicts the drift of six randomly selected hidden layer units across 30 cycles in Room 1.

bioRxiv preprint doi: https://doi.org/10.1101/2024.08.11.607484; this version posted January 29, 2025. The copyright holder for this preprint (which was not certified by peer review) is the author/funder, who has granted bioRxiv a license to display the preprint in perpetuity. It is made available under aCC-BY 4.0 International license.

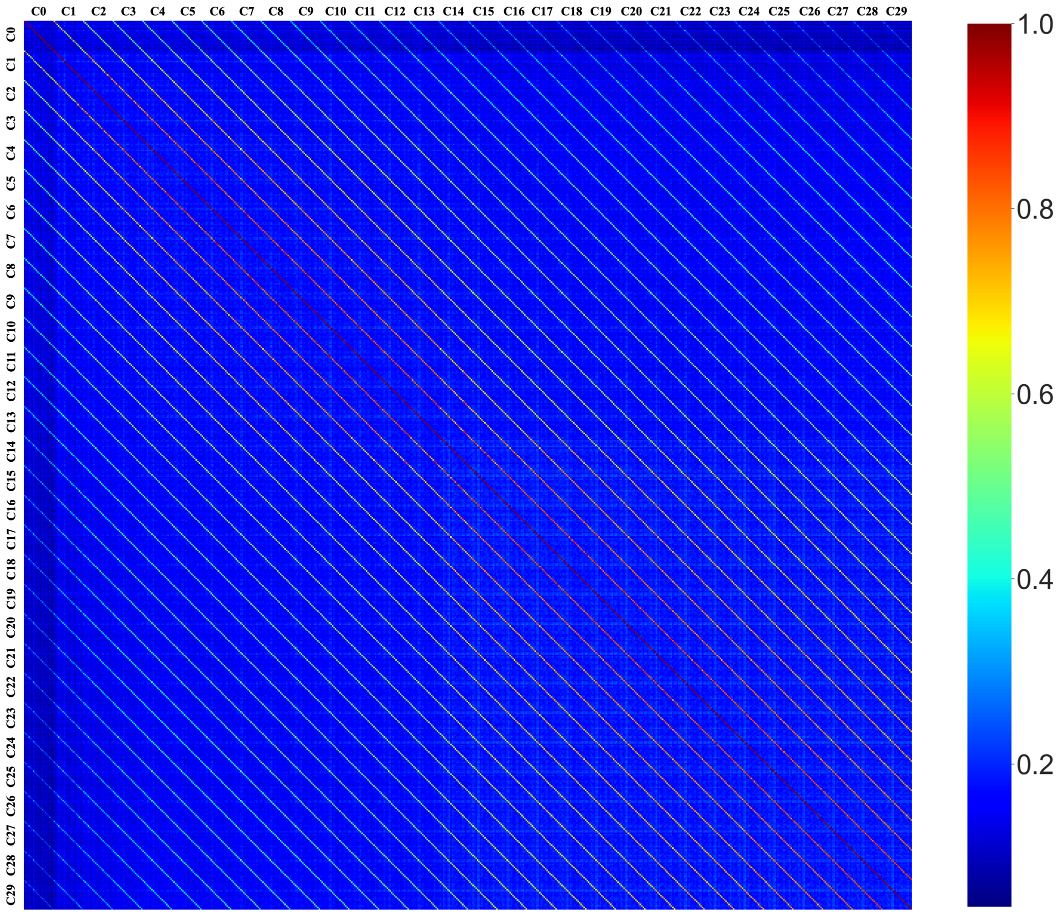

Figure 1: Cross correlation of all 600 recorded trials. Each pixel represents a comparison of two trials. During each cycle, the sequence in which the agent explores the 20 rooms is shuffled. We re-organize the room sequence when plotting the cross-correlation between trials to ease visualization. The periodic lines indicate a strong correlation of spatial representations generated when the animals entered the same room, even in different cycles.

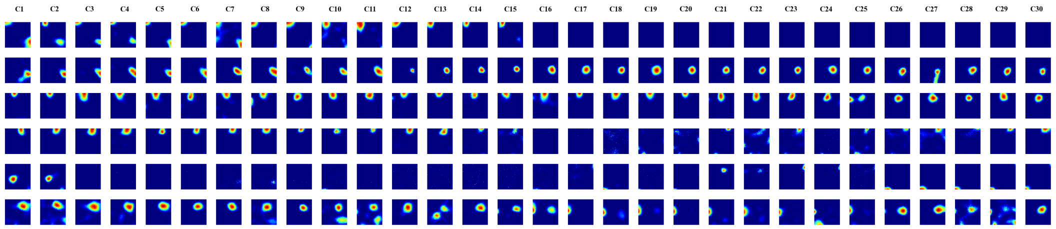

Figure 2: Illustration of place field drift across multiple visits to the same room over 30 cycles.

4 Additional results

4.1 Pattern completion results of RAE

We train the RAE to reconstruct the complete, noiseless experiences from partial and noisy sensory inputs. To examine pattern-completion performance, we pause training after each trial to record the reconstructed values and compute the average firing rate at each location for each output channel. This process is similar to the method used to gather firing profiles from the hidden layer. In Suppl. Fig. 3,

bioRxiv preprint doi: https://doi.org/10.1101/2024.08.11.607484; this version posted January 29, 2025. The copyright holder for this preprint (which was not certified by peer review) is the author/funder, who has granted bioRxiv a license to display the preprint in perpetuity. It is made available under aCC-BY 4.0 International license.

we plot 20 randomly selected WSM cells and their reconstructed values. All outputs accurately reflect the sensory changes appearing in the original WSM signals.

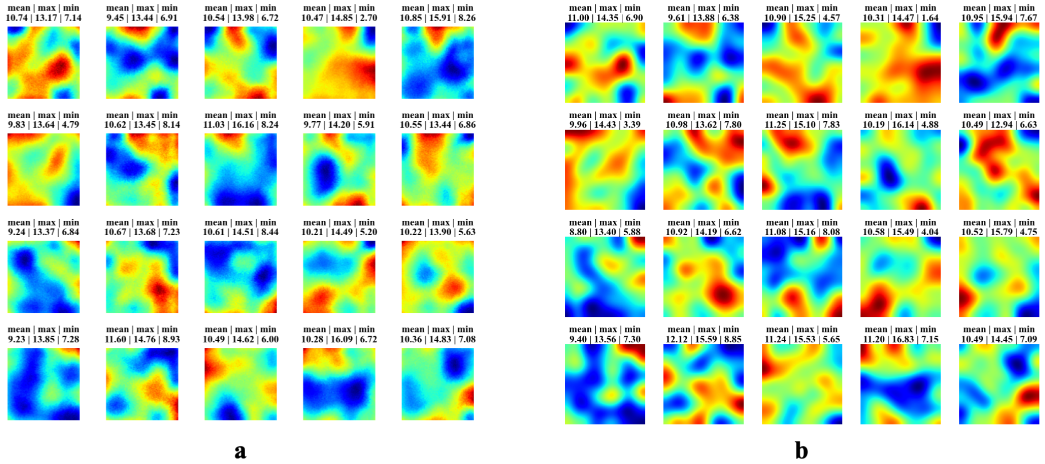

Figure 3: Example pattern completed WSM signals and their corresponding ground truths. **a.** 20 randomly selected WSM signals reconstructed by the RAE and plotted on the 2D space map. **b.** The ground truth values corresponding to the WSM cells in panel a.

4.2 Robustness to parameter variations

We have verified that the emergence of place cells is consistent across different environment shapes, for example triangles (Suppl. Fig. 4a) and circles (Suppl. Fig. 4b). In both cases, the hidden layer generated place-like patterns similar to those in the square rooms we used in our experiment.

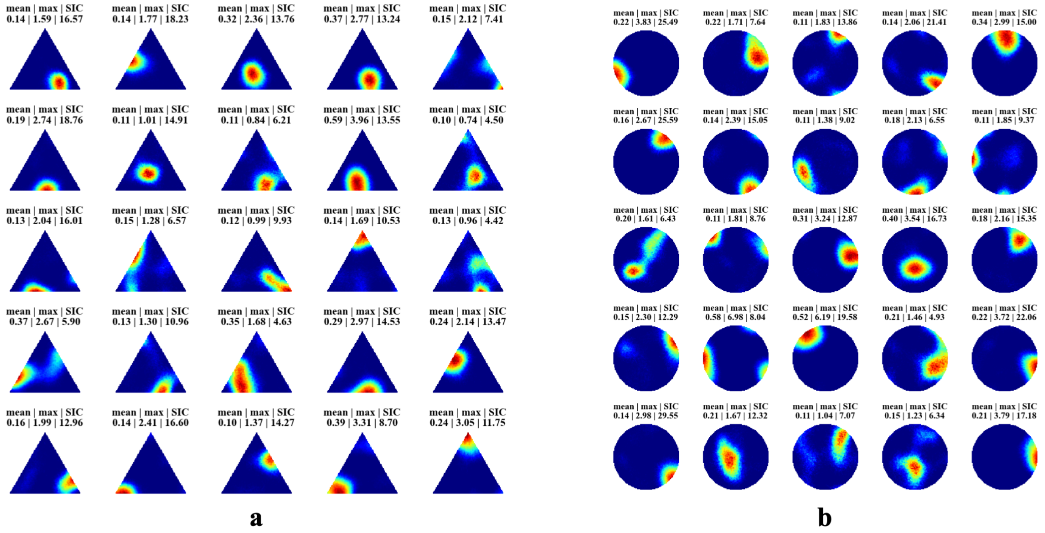

Figure 4: **a.** Example of emerged place-like patterns in a triangular room. **b.** Example of emerged place-like patterns in a circular room.

While we fixed the parameter values during our experiments to enhance reproducibility, we also verified that place cell emergence is robust to parameter changes and does not require specific values. To quantify, we evaluated the firing maps of hidden layer units using three metrics: (1) the percentage of active units, defined as units with a maximum firing rate > 0.1 Hz; (2) the percentage of place units, identified by a Spatial Information Content (SIC) > 5; (3) the average SIC across all active units.

bioRxiv preprint doi: https://doi.org/10.1101/2024.08.11.607484; this version posted January 29, 2025. The copyright holder for this preprint (which was not certified by peer review) is the author/funder, who has granted bioRxiv a license to display the preprint in perpetuity. It is made available under aCC-BY 4.0 International license.

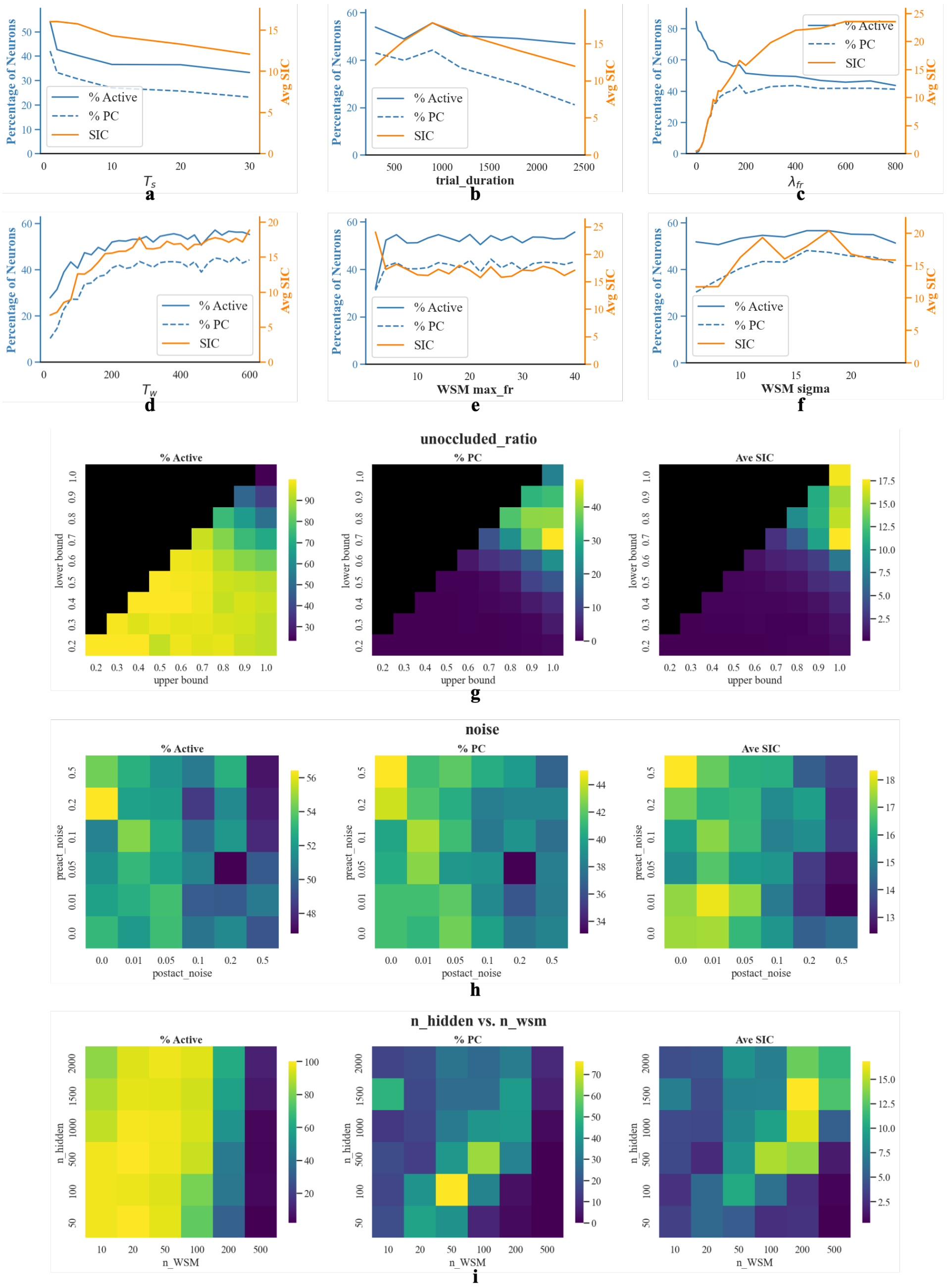

Figure 5: **a-i.** Impact of varying specific parameters, with others held constant at the default values, on place cell emergence. All experiments are conducted in a single trial room. Evaluation of hidden layer firing rate maps included three metrics: percentage of active cells (*max_fr* > 0.1 Hz) among all hidden units, percentage of place cells (*max_fr* > 0.1 Hz and SIC > 5) among all hidden units, and average SIC across active units. **a.** Duration of episodic memory segments. **b.** Total trial duration. **c.** Coefficient of firing rate loss. **d.** Warmup trial length (the recall length is also set to the same value). **e.** Maximum firing rate for each WSM channel. **f.** Sigma value for smoothing WSM signals. **g.** Ratio of unoccluded experience. We randomly preserve a fraction of the experience and train the RAE to recall the masked part, where the fraction is drawn uniformly in the interval $[r_{min}, r_{max}]$. Here we vary the bounds on this interval. **h.** Effects of pre-activation and post-activation noise. **i.** Co-varying the number of hidden units and the number of WSM cells (dimension of EV).

bioRxiv preprint doi: https://doi.org/10.1101/2024.08.11.607484; this version posted January 29, 2025. The copyright holder for this preprint (which was not certified by peer review) is the author/funder, who has granted bioRxiv a license to display the preprint in perpetuity. It is made available under aCC-BY 4.0 International license.

Figure 5 shows that: (1) Increasing the duration of each episodic memory segment slightly reduces the number of place cells, as longer episodes likely involve multiple locations, decreasing spatial specificity. Despite this, the majority of active cells continue to exhibit place-like characteristics. (see Fig. 5a). (2) The number of place cells decreases slowly as trial duration increases. This decrease is due to the optimizer forcing the network to encode WSMs more efficiently after the MSE loss stops decreasing. This optimized encoding is thus overfitted to one single room and requires individual cells to fire at unrealistic rates, which are unlikely to occur in biological systems (see Fig. 5b) (3) As λ_{fr} increases, all active cells become place cells. (4) The number of place cells and active cells increases as the recall length increases, stabilizing once the recall duration exceeds 200 seconds. (see Fig. 5d) (5) Neither the maximum firing rate of WSM signals nor the sigma value affects the emergence of PFs. (see Fig. 5e & 5f) (6) Place fields only emerge when the number of WSM signals exceeds 100, aligning with our hypothesis in the main text that the emergence of place fields requires a large number of WSM signals. We observed in our experiments that the number of hidden units required for PF emergence increases as the dimension of the experience vector (EV), i.e., the number of WSM signals, increases. This is likely because increasing the dimensionality of experience will increase the amount of information that must to be stored, and thereby require more hidden units. We leave exploration of the best ratio between input and hidden units for future work. Overall, our main finding is that the emergence of place fields is robust under a wide range of parameters.

4.3 Attractor landscape of the RAE model of place cells

Classical theories of CA3 episodic memory storage suggest that the recurrent connections establish attractor dynamics [29, 30, 28, 68]. Each attractor basin can be either a singular fixed point or a collection of points in the network's state space. Nearby network states evolve autonomously toward these attractors [47]. The attractor network theory of episodic memory also accounts for spatial memory storage, where each place-cell firing field corresponds to an attractor basin. Therefore, we also examined the attractor landscape of our RAE.

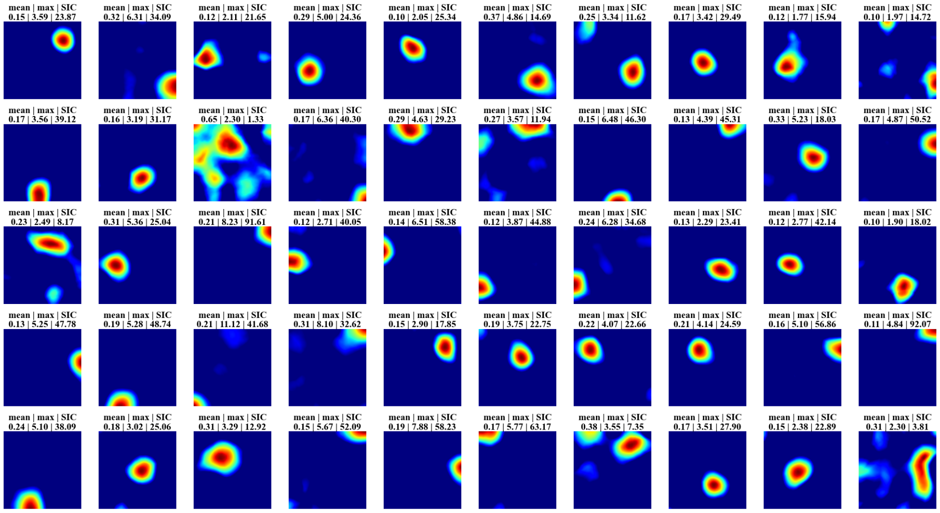

Figure 6: Example of neuronal responses at convergence points. For each location, we sample its specific EV, pause synaptic updates, and test the network by fixing this EV until convergence. Neuron states at each location are recorded upon convergence.

Attractor basins are locations where the network state becomes stable or dynamically stable. We thus examined how the network evolves when presented with a constant input. For this analysis, we simplified the network update function by removing the noise and bias terms:

$$\mathbf{v}_{t+1} = \Phi(\mathbf{v}_t) = (1-\alpha)\mathbf{v}_t + \alpha \left[W^{rc} f(\mathbf{v}_t) + W^{in} \mathbf{e}^* \right] \tag{6}$$

where we removed the bias and noise terms to simplify the calculation. As we show below, the network state update function is a contraction, and if inputs are held constant, the network will converge to a stable state. Given this property, we can plot the attractor landscape of our RAE

bioRxiv preprint doi: https://doi.org/10.1101/2024.08.11.607484; this version posted January 29, 2025. The copyright holder for this preprint (which was not certified by peer review) is the author/funder, who has granted bioRxiv a license to display the preprint in perpetuity. It is made available under aCC-BY 4.0 International license.

network by testing the network with location-specific EVs, and waiting for convergence to the attractor at each point.

As depicted in Suppl. Fig. 6, individual units maintain place-like patterns after the network has converged. This single-unit level continuous response at convergence will also result in a continuous landscape after the network converges. Thus, our network also aligns with the classical attractor theory of place cells.

Proof. If the input to the RNN is fixed (i.e., $\forall i, j \in [0, T], i \neq j$, we have $\mathbf{e}_i = \mathbf{e}_j = \mathbf{e}^*$), where T is the length of the input sequence. We would like to prove the network update function is a contraction, i.e., the network will converge to a fixed state. We work with the network update function:

$$\mathbf{v}_{t+1} = \Phi(\mathbf{v}_t) = (1-\alpha)\mathbf{v}_t + \alpha\left[W^{rc}f(\mathbf{v}_t) + W^{in}\mathbf{e}^*\right] . \tag{7}$$

To show that the network will converge to a fixed state, we first show that the network update function is a contraction. To do so, we need to show $\forall i, j \in [0, T], i \neq j$, if $\|\Phi(\mathbf{v}_j) - \Phi(\mathbf{v}_i)\| < k\|\mathbf{v}_j - \mathbf{v}_i\|$, where $k < 1$.

Suppose $\mathbf{v}_j - \mathbf{v}_i = \mathbf{u}$, then

$$\Phi(\mathbf{v}_j) = \Phi(\mathbf{v}_i + \mathbf{u}) = (1-\alpha)(\mathbf{v}_i + \mathbf{u}) + \alpha\left[W^{rc}f(\mathbf{v}_i + \mathbf{u}) + W^{in}\mathbf{e}^*\right] \tag{8}$$

$$\begin{aligned}\Phi(\mathbf{v}_j) - \Phi(\mathbf{v}_i) &= (1-\alpha)\mathbf{v}_j + \alpha\left[W^{rc}f(\mathbf{v}_j) + W^{in}\mathbf{e}^*\right] - (1-\alpha)\mathbf{v}_i - \alpha\left[W^{rc}f(\mathbf{v}_i) + W^{in}\mathbf{e}^*\right] \\ &= (1-\alpha)\mathbf{u} + \alpha W^{rc}\left[f(\mathbf{v}_i + \mathbf{u}) - f(\mathbf{v}_i)\right]\end{aligned} \tag{9}$$

By the triangle inequality and submultiplicativity,

$$\|\Phi(\mathbf{v}_j) - \Phi(\mathbf{v}_i)\| \leq (1-\alpha)\|\mathbf{u}\| + \alpha\|W^{rc}\|\|f(\mathbf{v}_i + \mathbf{u}) - f(\mathbf{v}_i)\| \tag{10}$$

Furthermore, if the activation function is bounded and is Lipschitz continuous on real numbers, a Lipschitz constant L exists such that $\|f(\mathbf{v}_i + \mathbf{u}) - f(\mathbf{v}_i)\|$ is bounded by $L\|\mathbf{u}\|$. Thus,

$$\begin{aligned}\|\Phi(\mathbf{v}_j) - \Phi(\mathbf{v}_i)\| &\leq (1-\alpha)\|\mathbf{u}\| + \alpha\|W^{rc}\|L\|\mathbf{u}\| \\ &= (1 - \alpha + \alpha\|W^{rc}\|L)\|\mathbf{u}\| = k\|\mathbf{u}\|\end{aligned} \tag{11}$$

The update function is a contraction mapping if $k = (1-\alpha) + \alpha\|W^{rc}\|L < 1$. Therefore, $\|W^{rc}\| < L^{-1}$ to ensure $k < 1$. The Lipschitz constant is $L = 0.25$ for sigmoid activation, $L = 0.5$ for tanh activation, and $L = 1$ for the positive part of ReLU activation.

Because the neuronal activation potentials are real numbers and we are using the distance function as the metric, the state-space is complete. By the Banach fixed-point theorem, iteratively applying this transformation will converge to a fixed point $\mathbf{v}^*$ in the state space. □

bioRxiv preprint doi: https://doi.org/10.1101/2024.08.11.607484; this version posted January 29, 2025. The copyright holder for this preprint (which was not certified by peer review) is the author/funder, who has granted bioRxiv a license to display the preprint in perpetuity. It is made available under aCC-BY 4.0 International license.

NeurIPS Paper Checklist

1. **Claims**

   Question: Do the main claims made in the abstract and introduction accurately reflect the paper's contributions and scope?

   Answer: [Yes]

   Justification: There are two claims in the abstract: 1) Training our autoencoder to pattern-complete and reconstruct sensory experiences with a constraint on total activity causes place cells to emerge in the encoding layer. 2) The emergent place fields reproduce key aspects of hippocampal phenomenology, including remapping, orthogonality of spatial representations in different rooms, robust place field emergence in differently shaped rooms, and slow representational drift of place fields. Both our conceptual framework and the results supporting the claims made in the abstract have been reported in the paper.

   Guidelines:

   - The answer NA means that the abstract and introduction do not include the claims made in the paper.
   - The abstract and/or introduction should clearly state the claims made, including the contributions made in the paper and important assumptions and limitations. A No or NA answer to this question will not be perceived well by the reviewers.
   - The claims made should match theoretical and experimental results, and reflect how much the results can be expected to generalize to other settings.
   - It is fine to include aspirational goals as motivation as long as it is clear that these goals are not attained by the paper.

2. **Limitations**

   Question: Does the paper discuss the limitations of the work performed by the authors?

   Answer: [Yes]

   Justification: We have discussed the limitation of this framework in the second to the last paragraph. The two major limitations are: 1) This paper could benefit from additional statistical comparison with rodent place cells. 2) We assumed the inherent structure of sensory signals during spatial navigation can be modeled by smooth Gaussian random fields. While this is supported by the evidence of abundant WSM cells in the medial entorhinal cortex, we invite future discussion on what composes sensory signals as well as systematic analyses on how to characterize their structures.

   Guidelines:

   - The answer NA means that the paper has no limitation while the answer No means that the paper has limitations, but those are not discussed in the paper.
   - The authors are encouraged to create a separate "Limitations" section in their paper.
   - The paper should point out any strong assumptions and how robust the results are to violations of these assumptions (e.g., independence assumptions, noiseless settings, model well-specification, asymptotic approximations only holding locally). The authors should reflect on how these assumptions might be violated in practice and what the implications would be.
   - The authors should reflect on the scope of the claims made, e.g., if the approach was only tested on a few datasets or with a few runs. In general, empirical results often depend on implicit assumptions, which should be articulated.
   - The authors should reflect on the factors that influence the performance of the approach. For example, a facial recognition algorithm may perform poorly when image resolution is low or images are taken in low lighting. Or a speech-to-text system might not be used reliably to provide closed captions for online lectures because it fails to handle technical jargon.
   - The authors should discuss the computational efficiency of the proposed algorithms and how they scale with dataset size.
   - If applicable, the authors should discuss possible limitations of their approach to address problems of privacy and fairness.

bioRxiv preprint doi: https://doi.org/10.1101/2024.08.11.607484; this version posted January 29, 2025. The copyright holder for this preprint (which was not certified by peer review) is the author/funder, who has granted bioRxiv a license to display the preprint in perpetuity. It is made available under aCC-BY 4.0 International license.

- While the authors might fear that complete honesty about limitations might be used by reviewers as grounds for rejection, a worse outcome might be that reviewers discover limitations that aren't acknowledged in the paper. The authors should use their best judgment and recognize that individual actions in favor of transparency play an important role in developing norms that preserve the integrity of the community. Reviewers will be specifically instructed to not penalize honesty concerning limitations.

3. **Theory Assumptions and Proofs**

   Question: For each theoretical result, does the paper provide the full set of assumptions and a complete (and correct) proof?

   Answer: [Yes]

   Justification: We have made the following assumptions: 1) sensory signals during spatial navigation are weakly spatial modulated. 2) sensory signals from various cortical regions compose high-dimensional vectors of experiences, and a brief, continuous sequence of experiences constructs a 'piece' of episodic memory. 3) Hippocampus CA3 can be modeled as recurrent autoencoders that autoencodes episodic memories. While these assumptions are challenging to test directly in in-vivo brains, we provide support to these assumptions with the experimental results from an extensive body of literature.

   Guidelines:

   - The answer NA means that the paper does not include theoretical results.
   - All the theorems, formulas, and proofs in the paper should be numbered and cross-referenced.
   - All assumptions should be clearly stated or referenced in the statement of any theorems.
   - The proofs can either appear in the main paper or the supplemental material, but if they appear in the supplemental material, the authors are encouraged to provide a short proof sketch to provide intuition.
   - Inversely, any informal proof provided in the core of the paper should be complemented by formal proofs provided in appendix or supplemental material.
   - Theorems and Lemmas that the proof relies upon should be properly referenced.

4. **Experimental Result Reproducibility**

   Question: Does the paper fully disclose all the information needed to reproduce the main experimental results of the paper to the extent that it affects the main claims and/or conclusions of the paper (regardless of whether the code and data are provided or not)?

   Answer: [Yes]

   Justification: We have outlined the detailed structure of our network implementations and experimental settings. All parameters are reported in either the main text or the supplemental materials.

   Guidelines:

   - The answer NA means that the paper does not include experiments.
   - If the paper includes experiments, a No answer to this question will not be perceived well by the reviewers: Making the paper reproducible is important, regardless of whether the code and data are provided or not.
   - If the contribution is a dataset and/or model, the authors should describe the steps taken to make their results reproducible or verifiable.
   - Depending on the contribution, reproducibility can be accomplished in various ways. For example, if the contribution is a novel architecture, describing the architecture fully might suffice, or if the contribution is a specific model and empirical evaluation, it may be necessary to either make it possible for others to replicate the model with the same dataset, or provide access to the model. In general. releasing code and data is often one good way to accomplish this, but reproducibility can also be provided via detailed instructions for how to replicate the results, access to a hosted model (e.g., in the case of a large language model), releasing of a model checkpoint, or other means that are appropriate to the research performed.

bioRxiv preprint doi: https://doi.org/10.1101/2024.08.11.607484; this version posted January 29, 2025. The copyright holder for this preprint (which was not certified by peer review) is the author/funder, who has granted bioRxiv a license to display the preprint in perpetuity. It is made available under aCC-BY 4.0 International license.

- While NeurIPS does not require releasing code, the conference does require all submissions to provide some reasonable avenue for reproducibility, which may depend on the nature of the contribution. For example
  (a) If the contribution is primarily a new algorithm, the paper should make it clear how to reproduce that algorithm.
  (b) If the contribution is primarily a new model architecture, the paper should describe the architecture clearly and fully.
  (c) If the contribution is a new model (e.g., a large language model), then there should either be a way to access this model for reproducing the results or a way to reproduce the model (e.g., with an open-source dataset or instructions for how to construct the dataset).
  (d) We recognize that reproducibility may be tricky in some cases, in which case authors are welcome to describe the particular way they provide for reproducibility. In the case of closed-source models, it may be that access to the model is limited in some way (e.g., to registered users), but it should be possible for other researchers to have some path to reproducing or verifying the results.

5. **Open access to data and code**

   Question: Does the paper provide open access to the data and code, with sufficient instructions to faithfully reproduce the main experimental results, as described in supplemental material?

   Answer: [Yes]

   The code for replication is available at: `https://zhaozewang.github.io/projects/time_makes_space`

   Guidelines:

   - The answer NA means that paper does not include experiments requiring code.
   - Please see the NeurIPS code and data submission guidelines (`https://nips.cc/public/guides/CodeSubmissionPolicy`) for more details.
   - While we encourage the release of code and data, we understand that this might not be possible, so "No" is an acceptable answer. Papers cannot be rejected simply for not including code, unless this is central to the contribution (e.g., for a new open-source benchmark).
   - The instructions should contain the exact command and environment needed to run to reproduce the results. See the NeurIPS code and data submission guidelines (`https://nips.cc/public/guides/CodeSubmissionPolicy`) for more details.
   - The authors should provide instructions on data access and preparation, including how to access the raw data, preprocessed data, intermediate data, and generated data, etc.
   - The authors should provide scripts to reproduce all experimental results for the new proposed method and baselines. If only a subset of experiments are reproducible, they should state which ones are omitted from the script and why.
   - At submission time, to preserve anonymity, the authors should release anonymized versions (if applicable).
   - Providing as much information as possible in supplemental material (appended to the paper) is recommended, but including URLs to data and code is permitted.

6. **Experimental Setting/Details**

   Question: Does the paper specify all the training and test details (e.g., data splits, hyperparameters, how they were chosen, type of optimizer, etc.) necessary to understand the results?

   Answer: [Yes]

   Justification: In the main text, we have detailed the parameters and methods used to implement the recurrent autoencoder and replicate the experiments. Furthermore, we have comprehensively included every single detail necessary for reproducing our experiments in the supplemental materials.

   Guidelines:

bioRxiv preprint doi: https://doi.org/10.1101/2024.08.11.607484; this version posted January 29, 2025. The copyright holder for this preprint (which was not certified by peer review) is the author/funder, who has granted bioRxiv a license to display the preprint in perpetuity. It is made available under aCC-BY 4.0 International license.

- The answer NA means that the paper does not include experiments.
- The experimental setting should be presented in the core of the paper to a level of detail that is necessary to appreciate the results and make sense of them.
- The full details can be provided either with the code, in appendix, or as supplemental material.

7. **Experiment Statistical Significance**

   Question: Does the paper report error bars suitably and correctly defined or other appropriate information about the statistical significance of the experiments?

   Answer: [Yes]

   Justification: We have reported the results with standard deviations in our main text where statistical details are significant to the claims we made. For plots, all values are indicated, thus error bars have not been included.

   Guidelines:

   - The answer NA means that the paper does not include experiments.
   - The authors should answer "Yes" if the results are accompanied by error bars, confidence intervals, or statistical significance tests, at least for the experiments that support the main claims of the paper.
   - The factors of variability that the error bars are capturing should be clearly stated (for example, train/test split, initialization, random drawing of some parameter, or overall run with given experimental conditions).
   - The method for calculating the error bars should be explained (closed form formula, call to a library function, bootstrap, etc.)
   - The assumptions made should be given (e.g., Normally distributed errors).
   - It should be clear whether the error bar is the standard deviation or the standard error of the mean.
   - It is OK to report 1-sigma error bars, but one should state it. The authors should preferably report a 2-sigma error bar than state that they have a 96% CI, if the hypothesis of Normality of errors is not verified.
   - For asymmetric distributions, the authors should be careful not to show in tables or figures symmetric error bars that would yield results that are out of range (e.g. negative error rates).
   - If error bars are reported in tables or plots, The authors should explain in the text how they were calculated and reference the corresponding figures or tables in the text.

8. **Experiments Compute Resources**

   Question: For each experiment, does the paper provide sufficient information on the computer resources (type of compute workers, memory, time of execution) needed to reproduce the experiments?

   Answer: [Yes]

   Justification: We have included the computer resources we used in the Supplemental materials. We didn't include this information in the main text as we have verified that our results do not depend on computational resources to replicate. Both CPU and GPU are sufficient for our proposed experiments.

   Guidelines:

   - The answer NA means that the paper does not include experiments.
   - The paper should indicate the type of compute workers CPU or GPU, internal cluster, or cloud provider, including relevant memory and storage.
   - The paper should provide the amount of compute required for each of the individual experimental runs as well as estimate the total compute.
   - The paper should disclose whether the full research project required more compute than the experiments reported in the paper (e.g., preliminary or failed experiments that didn't make it into the paper).

9. **Code Of Ethics**

bioRxiv preprint doi: https://doi.org/10.1101/2024.08.11.607484; this version posted January 29, 2025. The copyright holder for this preprint (which was not certified by peer review) is the author/funder, who has granted bioRxiv a license to display the preprint in perpetuity. It is made available under aCC-BY 4.0 International license.

Question: Does the research conducted in the paper conform, in every respect, with the NeurIPS Code of Ethics `https://neurips.cc/public/EthicsGuidelines`?

Answer: [Yes]

Justification: Yes, our research conforms with the NeurIPS Code of Ethics in every respect.

- The answer NA means that the authors have not reviewed the NeurIPS Code of Ethics.
- If the authors answer No, they should explain the special circumstances that require a deviation from the Code of Ethics.
- The authors should make sure to preserve anonymity (e.g., if there is a special consideration due to laws or regulations in their jurisdiction).

10. **Broader Impacts**

Question: Does the paper discuss both potential positive societal impacts and negative societal impacts of the work performed?

Answer: [NA]

Justification: There is no societal impact of the work performed

Guidelines:

- The answer NA means that there is no societal impact of the work performed.
- If the authors answer NA or No, they should explain why their work has no societal impact or why the paper does not address societal impact.
- Examples of negative societal impacts include potential malicious or unintended uses (e.g., disinformation, generating fake profiles, surveillance), fairness considerations (e.g., deployment of technologies that could make decisions that unfairly impact specific groups), privacy considerations, and security considerations.
- The conference expects that many papers will be foundational research and not tied to particular applications, let alone deployments. However, if there is a direct path to any negative applications, the authors should point it out. For example, it is legitimate to point out that an improvement in the quality of generative models could be used to generate deepfakes for disinformation. On the other hand, it is not needed to point out that a generic algorithm for optimizing neural networks could enable people to train models that generate Deepfakes faster.
- The authors should consider possible harms that could arise when the technology is being used as intended and functioning correctly, harms that could arise when the technology is being used as intended but gives incorrect results, and harms following from (intentional or unintentional) misuse of the technology.
- If there are negative societal impacts, the authors could also discuss possible mitigation strategies (e.g., gated release of models, providing defenses in addition to attacks, mechanisms for monitoring misuse, mechanisms to monitor how a system learns from feedback over time, improving the efficiency and accessibility of ML).

11. **Safeguards**

Question: Does the paper describe safeguards that have been put in place for responsible release of data or models that have a high risk for misuse (e.g., pretrained language models, image generators, or scraped datasets)?

Answer: [NA]

Justification: This paper does not pose such risks.

Guidelines:

- The answer NA means that the paper poses no such risks.
- Released models that have a high risk for misuse or dual-use should be released with necessary safeguards to allow for controlled use of the model, for example by requiring that users adhere to usage guidelines or restrictions to access the model or implementing safety filters.
- Datasets that have been scraped from the Internet could pose safety risks. The authors should describe how they avoided releasing unsafe images.

bioRxiv preprint doi: https://doi.org/10.1101/2024.08.11.607484; this version posted January 29, 2025. The copyright holder for this preprint (which was not certified by peer review) is the author/funder, who has granted bioRxiv a license to display the preprint in perpetuity. It is made available under aCC-BY 4.0 International license.

- We recognize that providing effective safeguards is challenging, and many papers do not require this, but we encourage authors to take this into account and make a best faith effort.

12. **Licenses for existing assets**

    Question: Are the creators or original owners of assets (e.g., code, data, models), used in the paper, properly credited and are the license and terms of use explicitly mentioned and properly respected?

    Answer: [NA]

    Justification: All assets are created by ourselves.

    Guidelines:

    - The answer NA means that the paper does not use existing assets.
    - The authors should cite the original paper that produced the code package or dataset.
    - The authors should state which version of the asset is used and, if possible, include a URL.
    - The name of the license (e.g., CC-BY 4.0) should be included for each asset.
    - For scraped data from a particular source (e.g., website), the copyright and terms of service of that source should be provided.
    - If assets are released, the license, copyright information, and terms of use in the package should be provided. For popular datasets, `paperswithcode.com/datasets` has curated licenses for some datasets. Their licensing guide can help determine the license of a dataset.
    - For existing datasets that are re-packaged, both the original license and the license of the derived asset (if it has changed) should be provided.
    - If this information is not available online, the authors are encouraged to reach out to the asset's creators.

13. **New Assets**

    Question: Are new assets introduced in the paper well documented and is the documentation provided alongside the assets?

    Answer: [NA]

    Justification: Our paper does not release new assets.

    Guidelines:

    - The answer NA means that the paper does not release new assets.
    - Researchers should communicate the details of the dataset/code/model as part of their submissions via structured templates. This includes details about training, license, limitations, etc.
    - The paper should discuss whether and how consent was obtained from people whose asset is used.
    - At submission time, remember to anonymize your assets (if applicable). You can either create an anonymized URL or include an anonymized zip file.

14. **Crowdsourcing and Research with Human Subjects**

    Question: For crowdsourcing experiments and research with human subjects, does the paper include the full text of instructions given to participants and screenshots, if applicable, as well as details about compensation (if any)?

    Answer: or [NA]

    Justification: Our paper does not involve crowdsourcing nor research with human subjects.

    Guidelines:

    - The answer NA means that the paper does not involve crowdsourcing nor research with human subjects.
    - Including this information in the supplemental material is fine, but if the main contribution of the paper involves human subjects, then as much detail as possible should be included in the main paper.

bioRxiv preprint doi: https://doi.org/10.1101/2024.08.11.607484; this version posted January 29, 2025. The copyright holder for this preprint (which was not certified by peer review) is the author/funder, who has granted bioRxiv a license to display the preprint in perpetuity. It is made available under aCC-BY 4.0 International license.

- According to the NeurIPS Code of Ethics, workers involved in data collection, curation, or other labor should be paid at least the minimum wage in the country of the data collector.

15. **Institutional Review Board (IRB) Approvals or Equivalent for Research with Human Subjects**

    Question: Does the paper describe potential risks incurred by study participants, whether such risks were disclosed to the subjects, and whether Institutional Review Board (IRB) approvals (or an equivalent approval/review based on the requirements of your country or institution) were obtained?

    Answer: [NA]

    Justification: Our paper does not involve crowdsourcing nor research with human subjects.

    Guidelines:

    - The answer NA means that the paper does not involve crowdsourcing nor research with human subjects.
    - Depending on the country in which research is conducted, IRB approval (or equivalent) may be required for any human subjects research. If you obtained IRB approval, you should clearly state this in the paper.
    - We recognize that the procedures for this may vary significantly between institutions and locations, and we expect authors to adhere to the NeurIPS Code of Ethics and the guidelines for their institution.
    - For initial submissions, do not include any information that would break anonymity (if applicable), such as the institution conducting the review.
